# Health-Promoting Properties of the Wild-Harvested Meat of Roe Deer (*Capreolus capreolus* L.) and Red Deer (*Cervus elaphus* L.)

**DOI:** 10.3390/ani11072108

**Published:** 2021-07-15

**Authors:** Anna Milczarek, Alina Janocha, Grażyna Niedziałek, Michalina Zowczak-Romanowicz, Elżbieta Horoszewicz, Sławomir Piotrowski

**Affiliations:** Faculty of Agrobioengineering and Animal Husbandry, Siedlce University of Natural Sciences and Humanities, Institute of Animal Science and Fisheries, Bolesława Prusa 14, 08-110 Siedlce, Poland; alina.janocha@uph.edu.pl (A.J.); grazyna.niedzialek@uph.edu.pl (G.N.); michalina.zowczak@gmail.com (M.Z.-R.); elzbieta.horoszewicz@uph.edu.pl (E.H.); Piotrowskislawek@wp.pl (S.P.)

**Keywords:** roe and red deer meat, proximate composition, fatty acids, Fe, Cd, Pb

## Abstract

**Simple Summary:**

Due to its chemical composition, roe and red deer meat can be a valuable component of a well-balanced diet for the modern consumer; however, the raw material from the carcasses of animals living in the wild can show differences in nutritional value. The present study provides an analysis of the content of basic nutrients (protein, fat, crude ash), Fe, Cd and Pb and fatty acid profile based on which the health-promoting value of wild-harvested roe deer and red deer meat was evaluated. Sixty carcasses were selected for chemical analysis; 30 of roe deer (15 does and 15 bucks) and 30 of red deer (15 hinds and 15 stags). The study leads to the conclusion that the evaluated meat of roe deer and red deer had high dietary value as it contained a high protein and low fat content and had an advantageous fatty acids composition. The noted content of cadmium and lead in the haunch of roe and red deer was significantly lower than permissible standard values, which corroborates that the meat is healthy and safe for consumers.

**Abstract:**

The present studies aimed to analyse and assess the health-promoting properties of the *musculus semimembranosus* (MS) of roe and red deer harvested in the wild. The experimental materials comprising the carcasses of roe deer (15 does and 15 bucks) and red deer (15 hinds and 15 stags) were selected for analysis based on the following criteria: age of animals at harvest—3–4 years; time that passed from the harvest of animals to carcass cutting—48–54 h. After chilling the carcasses for 24 h at +2 °C, the haunches were cut from the carcasses and transported to the laboratory in isothermal ice-packed containers. Samples of the *musculus semimembranosus* were analysed to determine the chemical composition (proximate components, energy value, Fe, Pb, Cd, fatty acids). More (*p* ≤ 0.05) dry matter and total protein and less (*p* ≤ 0.05) crude fat was found in the *musculus semimembranosus* of roe deer in comparison to red deer. No significant influence of the animal’s sex was observed in the content of the evaluated nutrients, apart from crude fat content, which was increased in the haunch of females from both species. The energy content in the muscle of roe deer was 10% higher than the energy value of MS in red deer (*p* ≤ 0.05). The content of iron was significantly (*p* ≤ 0.05) higher (6.64 mg/kg) in the meat of red deer compared to the meat of roe deer (31.68 mg/kg). Roe deer haunch contained more lead but less cadmium than red deer haunch. Irrespective of sex, the lipid fraction of red deer muscle contained more saturated fatty acids (SFA). Intramuscular fat (IMF) in roe deer meat contained a higher percentage of polyunsaturated fatty acids (PUFA). The atherogenicity (AI) and thrombogenicity (TI) index values were significantly lower in roe deer haunch, and the hypocholesterolaemic-to-hypercholesterolaemic fatty acids ratio (HH) was lower (*p* ≤ 0.05) in red deer meat. To sum up, the evaluated roe deer and red deer haunch featured high dietary value as it contained a high protein and low fat content and had an advantageous fatty acids composition. As regards the content of cadmium and lead, roe deer and red deer haunch can be considered safe for consumers.

## 1. Introduction

In the past hunting was a traditional activity of the population in the rural environment. Contemporary hunting is, in the first place, a form of nature conservation aimed at adapting the number of animals living in the wild with the changing environment [1]. This is accompanied by acquiring very valuable meat which satisfies many requirements and expectations of consumers in terms of the nutritional value, attractive and unique sensory quality and healthiness of food products associated with low energy value [2,3,4,5].

Despite having numerous advantages, roe and red deer meat is not popular in the diet of Poles, who consume about 80 g of it per capita in a year, while the annual consumption of all meat is 75 kg/capita/year. Roe and red deer meat consumption in the EU ranges between 0.08 kg/capita/year (Poland and Portugal) and 5.7 kg/capita/year in France or up to 8.4 kg/capita/year for Andalusian hunters [6,7,8].

A review of reference literature [2,4,5] has shown that the quality of wild game meat is very high but also highly differentiated. This refers to both interspecies differences and intraspecies variations in the quality of meat. Several authors [9,10,11,12,13,14] reported that the nutritional value of game meat depends on many factors, including season of the year, environment, climate, sex and age. Numerous studies [15,16,17,18] revealed that wild game meat contains high complete protein and low fat levels with an advantageous fatty acids composition. The intramuscular fat (IMF) of wild animals has a desirable healthy PUFA/SFA and n-6/n-3 PUFAs ratios [15,19,20]. The aforementioned components of roe and red deer meat are bioactive ingredients recommended in preventing civilization diseases [15,21,22]. 

On the other hand, roe and red deer meat can pose a risk to consumers in view of the progressing degradation of the natural environment associated with the accumulation of heavy metals in wild game’s feed [23,24]. Although contamination due to agricultural practices of any kind can be easily prevented through imposing a ban or limitation on their use, it is much more difficult to reduce the emissions of heavy metals and their compounds. Heavy metal compounds are not biodegradable and do not decay. They are stored in plants and as such are consumed by animals. They accumulate both in tissues and body organs [25]. Analysis of meat harvested in forests and fields can be a bioindicator of the level of environmental contamination. By monitoring the existence of animals, the place of harvesting and many other factors, the level of environmental pollution can be assessed [25,26,27,28]. Evaluation of the degree of bioaccumulation of lead and cadmium in the muscle tissue of animals, including roe and red deer, is a criterion for assessing the safety of such products for consumption.

The study aimed to compare the proximate composition (protein, fat, crude ash), Fe, Cd and Pb and fatty acids profile based on which the health-promoting value of wild-harvested roe deer and red deer meat was evaluated.

## 2. Materials and Methods

### 2.1. Harvesting the Animals and Acquisition of Muscle Samples

The experimental materials comprised the carcasses of roe deer (*Capreolus capreolus* L.) and red deer (*Cervus elaphus* L.) hunter-harvested in the same forests of north-eastern Poland. The animals were shot in November and December. Among the carcasses supplied to the meat processing plant, 60 carcasses of roe deer (15 does and 15 bucks) and red deer (15 hinds and 15 stags) were selected for analysis, based on the following criteria: age of animals at harvest—3–4 years (the age of animals was estimated based on the wear of mandibular premolars and molars; [29]); time that passed from the harvesting of animals to carcass cutting—48–54 h. In the slaughterhouse all the hunted animals went through veterinary inspection. The average carcass weights of roe deer was 16–18 kg, and that of red deer—90–93 kg. After chilling the carcasses for 24 h at +2 °C, the haunches were cut from the carcasses. The *musculus semimembranosus* (MS) of each carcass was transported to the laboratory in isothermal containers with ice. In order to determine the chemical composition of MS, out of 60 collected samples of meat 20 test samples (*N* = 20) were selected taking the species and sex of the animals into account (*n* = 5).

### 2.2. Analysis

#### 2.2.1. Proximate Composition of Meat

The analysis of the proximate chemical composition of meat included determination of dry matter content (930.15), crude protein content by the Kjeldahl method (990.03), crude fat content by the Soxhlet method (991.36) and crude ash content (942.05) according to AOAC methodology [30]. The content of protein and water in the evaluated muscle of roe deer and red deer was used to calculate the water–protein ratio (W/P).

#### 2.2.2. Calculation of Energy Value

Energy value was calculated using individual energy factors for protein—4.00 kcal/g (16.78 kJ/g) and fat—9.00 kcal/g (37.62 kJ/g) [31].

#### 2.2.3. Cadmium, Lead, Iron

The content of cadmium (Cd), lead (Pb) and iron (Fe) was determined by atomic absorption spectrometry according to PN-EN 14084 [32]. The prepared samples were mineralised at elevated pressure in a MARS 5 Xpress microwave (CEM, Matthews, NC, USA), using 65% nitric acid (MERCK Suprapur; catalogue no. 1.00441; 0.01 dm^3^ per 0.5 g of the sample). Max. temperature was set to 200 °C, mineralisation time 40 min. As part of the quality control of the method, the Certified Reference Material NCS ZC 73009 (China National Analysis Center for Iron and Steel, Beijing, China) was tested. The mineralised samples were cooled and transferred quantitatively to 50 mL volumetric flasks. The flasks were then filled with deionised water to a volume of 50 mL. The content of Cd and Pb was determined in a graphite furnace with Zeeman background correction (in argon atmosphere) using an AA 240Z Varian spectrometer (Agilent Technologies Inc., Santa Clara, CA, USA). The content of Fe was determined by atomic absorption spectrometry with an acetylene-air flame. The levels of elements in muscle were expressed in mg/kg of fresh mass, taking into account all quantitative processes during material preparation and reagent blanks. 

#### 2.2.4. Fatty Acids

The fatty acid composition of intramuscular fat in *musculus semimembranosus* was determined by gas chromatography. Fatty acid methyl esters (FAMEs) were analysed following fat extraction according to Folch [33]. Gas chromatographic (GC) analyses were performed using a Varian CG 3900 gas chromatograph with a flame ionisation detector (FID). FAMEs were separated in a CP Sil 88 capillary column, 100 m in length, inner diameter 0.25 mm, film thickness 0.25 μm. The analysis was carried out in increasing temperature conditions. The temperature program was as follows: 50 °C for 1 min, 30 °C/min up to 120 °C, 2 °C/min up to 160 °C, 30 min holding, 1 °C/min up to 200 °C, 5 °C/min up 250 °C, 1 min holding time. The temperature of the injector and the detector was 270 °C, the carrier gas (hydrogen) flow rate 2 mL/min, the size of the injected samples 1 μL, and the split ratio 1:50. Identification and quantification of FAMEs was based on retention times corresponding to reference mixtures (Supelco Inc., Bellefonte, PA, USA; Larodan AB, Solna, Sweden), using Star GC Workstation Version 5.5. software (Varian Inc., Walnut Creek, CA, USA). The following procedure was used to estimate the fatty acid composition as percentages of total fatty acids. The following groups of fatty acids were determined: saturated fatty acids (SFA), and unsaturated fatty acids (UFA), including monounsaturated fatty acids (MUFA) and polyunsaturated fatty acids (PUFA). Based on the percentage (%) of the total fatty acids, we calculated the atherogenic (AI) and thrombogenic (TI) indexes, as well as the hypocholesterolaemic-to-hypercholesterolaemic fatty acids ratio (HH) according to Ulbricht and Southgate [34] and Santos-Silva et al. [35]:

AI = (C12:0 + 4 × C14:0 + C16:0)/[ΣMUFA + Σ(n-6) + Σ(n-3)]

TI = (C14:0 + C16:0 + C18:0)/[0.5 × ΣMUFA + 0.5 × Σ(n-6) + 3 × Σ(n-3) + Σ(n-3)/Σ(n-6)]

HH = [(C18:1n-9 + C18:2n-6 + C20:4n-6 + C18:3n-3 + C20:5n-3 + C22:5n-3 + C22:6-n)/(C14:0 + C16:0)].

### 2.3. Statistical Analysis

The results were elaborated by statistical methods using two-way analysis of variance. The significance of differences between mean values was verified at the significance level α ≤ 0.05. The data was tested using post-hoc Tuckey’s test. The results were processed with STATISTICA PL 13.1 software [36].

## 3. Results

The analysis of proximate composition in the *musculus semimembranosus* showed more (*p* ≤ 0.05) dry matter and protein and less (*p* ≤ 0.05) fat in the muscle of roe deer compared to red deer (Table 1).

No significant influence of the animal’s sex was observed on the content of the evaluated nutrients, except for fat content, which was higher in the haunch of females irrespective of the species. Proteins in the muscles of red deer were significantly (by 14%) more hydrated (W/P ratio) than in that of roe deer. The only significant (*p* ≤ 0.05) influence noted was that of the species on the energy value of meat. The energy content in roe deer haunch was 10% higher than the energy value of red deer meat. 

Analysis of the content of iron (Table 2) in the haunch of wild game revealed that it was significantly (*p* ≤ 0.05) higher (by 6.64 mg/kg) in the meat of red deer than of roe deer (31.68 mg/kg). A higher content was determined in the meat of stags (41.07 mg/kg) than in that of hinds (35.43 mg/kg), while the findings were the reverse for roe deer (29.87 vs. 33.48 mg/kg); these differences were not significant.

In evaluating the level of lead and cadmium in the haunch of roe and red deer a significant relationship between the species and lead content was identified. Roe deer meat contained more lead in comparison to red deer meat, irrespective of sex. More (*p* > 0.05) cadmium was determined in the muscle of doe and stag than in that of buck and doe.

The studies noted a significant (*p* ≤ 0.05) influence of the species on the content of the determined saturated fatty acids, except C18:0, C20:0 and C22:0 (Table 3). 

In addition, a relationship (*p* ≤ 0.05) was discovered between the share of palmitic acid (C16:0) and lignoceric acid (C24:0) and the animal’s sex. Irrespective of sex, the lipid fraction of red deer muscle contained more saturated fatty acids (SFA).

Analysis of the percentage of respective monounsaturated fatty acids in MS showed a significant (*p* ≤ 0.05) influence of the species of animals on the content of acids, except C17:1 (Table 4). 

Both sex and species had a significant influence on the content of oleic acid in the haunch. Roe deer meat contained a significantly higher (by 2.377 percentage points) of MUFA compared to red deer.

Assessment of the level of respective polyunsaturated fatty acids revealed a significant (*p* ≤ 0.05) relationship between the species and their content, except C20:3 n-6 (Table 5). 

*Musculus semimembranosus* of females contained more (*p* ≤ 0.05) C20:3n-3 and C22:2 acids than that of males. The lipid fraction in roe deer meat contained a higher (by 2.244 percentage points) of PUFA. 

Significantly lower atherogenicity (AI) and thrombogenicity (TI) index values were noted in roe deer muscle (Table 6). 

However, the HH index was lower (*p* ≤ 0.05) in the meat of red deer compared to roe deer. The muscle of females of both roe and red deer featured a better (*p* ≤ 0.05) hypocholesterolaemic–hypercholersterolaemic fatty acids ratio (HH).

## 4. Discussion

A concentration of proximate components in the muscle tissue has an influence on the dietary quality of meat. Game meat in general has a low lipid content, typically less than 3%, in comparison to meat from livestock. In addition, lipids in muscles from game animals are dominated by structural lipids with very desirable fatty acids profiles and a low percentage of intramuscular fat (IMF) [37,38]. A higher content of dry mass, total protein and crude fat but less minerals in comparison to the results of our own study were revealed in red deer haunch by Daszkiewicz et al. [5]. In turn, Florek et al. [39] noted less proximate components in the dry mass of red deer muscle tissue. Our own results relating to higher IMF content in the meat of red deer hind vs. stag are consistent with the findings of Polak et al. [9], but contrary to those of Daszkiewicz et al. [40] who noted a reverse relationship—the muscle of hinds containing less fat than that of stags (0.56% vs. 0.96%). The dry matter content in the muscle of roe deer was lower than that previously indicated [41,42]. Winkelmayer et al. [42] reported that the roe deer venison contains on average 25.88–27.97% dry matter (*M. longissimus*); similarly, Zomborszky et al. [41] noted its dry matter content as 25.7% (*M. semimembranosus*) and 25.2% (*M. longissimus*). Dominik et al. [43] found a higher fat but lower protein content in *M. longissimus dorsi* (0.26% and 19.98%) and in *M. gluteus medius* (0.82% and 19.26%) of roe deer compared to our own results. Winkelmayer et al. [42] report the fat content in roe deer’s *M. longissimus* depending on the season of the year ranges from 0.36% (spring) to 1.78% (autumn). Analysing the influence of the animal’s sex on the content of protein and fat in roe deer meat, Daszkiewicz et al. [44] found more nutrients in the meat of does (22.79% and 1.46%) than of bucks (21.84% and 0.83%). Irrespective of where roe deer bucks were culled, Daszkiewicz et al. [45] noted a higher water-to-protein ratio than in our own study. In turn, the W/P ratio in red deer meat was close to values reported by Daszkiewicz et al. [5]. Daszkiewicz et al. [5] showed a water–protein (W/P) ratio from 3.29 to 3.56 in red deer meat, depending on the clearing and the culling place. In addition, the above-mentioned authors noted a higher energy value (395.20–413.7 kJ) of red deer meat in comparison to our own results. A reverse relationship for the energy value of roe deer meat was observed by Daszkiewicz et al. [44], as the meat of female roe deer had a higher energy value (437.50 kJ) than of male roe deer (397.63 kJ). 

The muscle tissue of animals is a source of easily assimilable iron and in game meat such iron is highly concentrated [15,46]. The mean (3.82 mg/100 g) content of iron in red deer meat determined in own studies was above the 3.07–3.34 mg/100 g range reported by Florek and Drozd [15]. Red deer meat can be a good source of Fe in the human diet, because—as reported by Purchas et al. [47]—beef from Charolais cattle contains 2.0 mg/100 g, and pork only 0.84–1.08 mg/100 g [48].

The leading toxic elements contaminating the environment are lead and cadmium [49,50]. Contamination of food with their compounds is largely related to the economic activity of humans, and their use in many industries, which is at the same time connected with the emission and accumulation of toxic elements in the environment [23,51]. Analysis of the content of lead and cadmium in the muscle tissue of roe deer and red deer showed a low level of these elements, not objectionable from a toxicological point of view. According to current legal regulations [52,53], the permissible content of lead in animal meat is 0.10 mg/kg, and of cadmium—0.05 mg/kg. The levels of cadmium and lead recorded in roe and red deer haunch were not dangerous for consumers’ health since the European Food Safety Authority (EFSA) indicates the permissible total weekly intake (PTWI) of cadmium as 2.5 µg/kg of body weight, and that of lead—25 µg/kg of body weight [54]. Srebočan et al. [55] observed 0.001–0.034 mg/kg of lead in roe deer meat, which was lower in comparison to our own results. In turn, Jarzyńska and Falandysz [56] and Skibniewski et al. [57] found that the levels of lead and cadmium were higher in red deer meat. In other studies [50,58], a higher (0.48–0.58 mg/kg) content of lead was observed in the meat of roe deer. According to Demesko et al. [28], a correlation exists between the content of heavy metals and the type of food and feeding place of roe deer and red deer. Gašparík et al. [25] report a wide range of lead content in the muscles of red deer—0.15–104.873 mg/kg. In studies [23,59,60] it is indicated that the content of lead in the muscle tissue of wild game hunted using lead ammunition depends on how far the wound is from the muscle. Taking this distance into account in the carcasses of wild game during treatment in meat processing plants ensures high quality, safety and healthiness of game meat. 

The composition and proportions of fatty acids in red meat are now widely discussed due to, among other things, their significant influence on human health [37,61]. Rule et al. [62] showed that wild animals have lower content of SFA 41%, ruminants (cattle and sheep) have higher content of 44–46%. C16:0 and C18:0 are predominant SFAs in red meat, which was also observed in our study. C16:0 as well as lauric acid (C12:0) and C14:0 which were found in considerably lower amounts exert atherogenic effects [10,17,63]. In turn, stearic acid (C18:0) and unsaturated fatty acids are classified as neutral and hypocholesterolaemic acids (DFA) in which human diet is often deficient [37]. Briggs et al. [64] claimed that SFAs should be replaced with monounsaturated fatty acids, in particular polyunsaturated fatty acids. 

Daszkiewicz and Mesinger [14] showed a lower content of hypercholesterolaemic ac-ids (OFA = C14:0 + C16:0) and a higher content of C18:0 in the meat of roe deer and red deer, which was corroborated by our own studies. The fatty acids profile of meat depends on, for instance, the breed and diet of animals. In the opinion of several authors [65,66], the observed favourable quantitative and qualitative proportions of fat in roe and red deer meat seem to be closely linked to an active lifestyle of the animals and a specific biodiversity of natural feeding grounds. Differences in the concentrations of individual UFAs in the IMF of does and hinds were reflected in differences between the average total content of MUFAs, PUFAs and UFAs, which was considerably higher in roe deer. In comparison to the results of our own studies, Daszkiewicz and Mesinger [17] showed an increase of 6.85 percentage points and 10.98 percentage points, while Strazdina et al. [67] a decrease of 5.14 and 5.22 percentage points in SFA in the meat of roe deer and red deer, respectively. A lower share of saturated fatty acids in the meat of female compared to male roe deer corroborated the results of Daszkiewicz et al. [44]. 

A content of PUFA in the meat of red deer and roe deer similar to our own results was observed by Strazdina et al. [67] and Triumf et al. [68]. In turn, Valencak and Gamsjäger [69] and Valencak et al. [19] report that PUFA account for as much as 65.4–66.3% in roe deer meat and 60.8–63.8% in red deer meat. A lower percentage of PUFA in the meat of female vs. male roe deer—10.88% vs. 18.28%—was noted by Daszkiewicz et al. [44]. Irrespective of sex, the share of PUFA reported by Daszkiewicz et al. [44] was considerably lower than that measured in own studies. 

Strazdina et al. [67], comparing the fatty acids composition of roe deer and red deer meat with that of other species of wild game and farm animals showed a higher content of n-6 and n-3 polyunsaturated fatty acids. In turn, Valencak et al. [19] found a higher share of PUFAn-3 in red deer and roe deer meat than in wild boar, hare and pheasant meat. A high share of PUFA when the level of saturated fatty acids is low has a beneficial effect on the dietary values of this meat, which is corroborated by AI, TI and HH indices. 

The AI and TI indices take into account the influence of respective fatty acids on human health, and particularly on the prevalence of atherosclerotic diseases. High index values are characteristic of meat with low dietary value [70]. The values of AI and TI were worse in red deer meat compared to roe deer meat. The atherogenicity index of roe deer and red deer meat corroborates the results of Strazdina et al. [67]. In our own studies no influence of the animal’s sex on the atherogenicity index value was noted, but Polak et al. [9] found that the animal’s sex had a significant influence on the AI of red deer’s *musculus semitendinosus*. 

The AI, TI and HH indices in the meat of roe deer and red deer corroborate its high dietary value as they are better than for beef [71] and pork [48].

## 5. Conclusions

The study leads to the conclusion that the evaluated *musculus semimembranosus* of roe and red deer had high dietary value because it contained a lot of protein and little fat and had an advantageous fatty acids composition. The noted content of cadmium and lead in the muscles of roe and red deer was significantly lower than permissible standard values, which corroborates that the meat is healthy and safe for consumers. To sum up, in view of its health-promoting properties, the meat of roe deer and red deer hunted in north-eastern Poland should be recommended for including in the human diet.

## Figures and Tables

**Table 1:** Proximate composition (%) and energy value (kJ/100 g) of *musculus semimembranosus* of roe deer and red deer.

Item	Sex	Species		$\overset{-}{x}$	*p*-Value			SEM
		Roe Deer	Red Deer		s	g	g x s	
Dry matter	Male	25.32	22.95	24.13	<0.05	0.674	0.578	0.210
	Female	24.86	22.61	23.73				
	$\overset{-}{x}$	25.09	22.78	23.93				
Crude protein	Male	24.19	21.75	22.97	<0.05	0.084	0.853	0.207
	Female	23.66	21.32	22.49				
	$\overset{-}{x}$	23.92	21.54	22.73				
Crude fat	Male	0.08 ^b^	0.11 ^b^	0.10	<0.05	<0.05	<0.05	0.010
	Female	0.11 ^b^	0.21 ^a^	0.16				
	$\overset{-}{x}$	0.10	0.16	0.13				
Crude ash	Male	1.05	1.08	1.06	0.734	0.498	0.324	0.011
	Female	1.09	1.07	1.08				
	$\overset{-}{x}$	1.07	1.08	1.07				
W/P	Male	3.10	3.55	3.32	<0.05	0.097	0.972	0.038
	Female	3.18	3.63	3.41				
	$\overset{-}{x}$	3.14	3.59	3.37				
Energy value	Male	408.88	369.24	389.06	<0.05	0.243	0.648	3.361
	Female	401.21	365.87	383.54				
	$\overset{-}{x}$	405.04	367.55	386.30				

s—species, g—sex, g x s—interaction sex x species, SEM—standard error of mean, W/B—water to protein ratio, ab—means with different superscripts are significantly different at *p* ≤ 0.05.

**Table 2:** Contents (mg/kg) iron, lead and cadmium of haunch.

Elements	Sex	Species		$\overset{-}{x}$	*p*-Value			SEM
		Roe Deer	Red Deer		s	g	g x s	
Fe	Male	29.87 ^b^	41.07 ^a^	35.47	<0.05	0.549	<0.05	0.980
	Female	33.48 ^b^	35.43 ^ab^	34.45				
	$\overset{-}{x}$	31.68	38.25	34.96				
Cd	Male	0.003	0.004	0.004	0.051	0.089	0.142	0.001
	Female	0.004	0.013	0.008				
	$\overset{-}{x}$	0.003	0.008	0.006				
Pb	Male	0.05	0.03	0.04	<0.05	0.058	0.224	0.005
	Female	0.03	0.02	0.03				
	$\overset{-}{x}$	0.04	0.03	0.04				

s—species, g—sex, g x s—interaction sex x species, SEM—standard error of mean, ab—means with different superscripts are significantly different at *p* ≤ 0.05.

**Table 3:** Saturated fatty acids profile (% total) of *musculus semimembranosus.*

Fatty Acids	Sex	Species		$\overset{-}{x}$	*p*-Value			SEM
		Roe Deer	Red Deer		s	g	g x s	
C12:0	Male	0.079	0.251	0.165	<0.05	0.129	0.365	0.018
	Female	0.095	0.314	0.205				
	$\overset{-}{x}$	0.087	0.283	0.185				
C14:0	Male	1.359	3.596	2.478	<0.05	0.397	0.973	0.183
	Female	1.159	3.411	2.285				
	$\overset{-}{x}$	1.259	3.503	2.381				
C15:0	Male	0.397	0.706	0.552	<0.05	0.068	0.055	0.036
	Female	0.393	0.886	0.639				
	$\overset{-}{x}$	0.395	0.796	0.596				
C16:0	Male	20.298	23.443	21.871	<0.05	<0.05	0.087	0.309
	Female	20.115	21.419	20.767				
	$\overset{-}{x}$	20.207	22.431	21.319				
C17:0	Male	1.078	0.735	0.907	<0.05	0.258	0.780	0.041
	Female	1.175	0.794	0.984				
	$\overset{-}{x}$	1.126	0.765	0.946				
C18:0	Male	19.477	19.065	19.183	0.719	0.655	0.227	0.237
	Female	19.107	19.865	19.779				
	$\overset{-}{x}$	19.292	19.465	19.378				
C20:0	Male	0.045	0.055	0.050	0.944	0.780	0.366	0.05
	Female	0.051	0.043	0.047				
	$\overset{-}{x}$	0.048	0.049	0.049				
C22:0	Male	0.031	0.028	0.030	0.104	0.187	0.213	0.004
	Female	0.031	0.006	0.018				
	$\overset{-}{x}$	0.031	0.017	0.024				
C24:0	Male	0.255	0.090	0.172	<0.05	<0.05	0.262	0.018
	Female	0.231	0.009	0.120				
	$\overset{-}{x}$	0.243	0.050	0.146				
SFA	Male	43.020	47.969	45.495	<0.05	0.252	0.733	0.504
	Female	42.356	46.747	44.552				
	$\overset{-}{x}$	42.688	47.358	45.023				

s—species, g—sex, g x s—interaction sex x species, SEM—standard error of mean, SFA—saturated fatty acids.

**Table 4:** Monounsaturated fatty acids profile (% total) of MS.

Fatty Acids	Sex	Species		$\overset{-}{x}$	*p*-Value			SEM
		Roe Deer	Red Deer		s	g	g x s	
C14:1	Male	0.375	1.529	0.952	<0.05	0.345	0.737	0.078
	Female	0.331	1.509	0.920				
	$\overset{-}{x}$	0.353	1.519	0.936				
C15:1	Male	0.036	0.075	0.056	<0.05	0.175	0.373	0.004
	Female	0.033	0.059	0.046				
	$\overset{-}{x}$	0.034	0.067	0.051				
C16:1	Male	1.967	6.410	4.189	<0.05	0.642	0.704	0.289
	Female	1.963	6.363	4.163				
	$\overset{-}{x}$	1.965	6.387	4.176				
C17:1	Male	0.340	0.348	0.344	0.550	0.550	0.860	0.009
	Female	0.348	0.363	0.355				
	$\overset{-}{x}$	0.344	0.355	0.350				
C18:1	Male	28.127 ^a^	19.736 ^c^	23.932	<0.05	<0.05	<0.05	0.534
	Female	28.075 ^a^	20.072 ^b^	24.074				
	$\overset{-}{x}$	28.101	19.904	24.003				
C20:1	Male	0.045	0.125	0.085	<0.05	0.361	0.611	0.006
	Female	0.059	0.129	0.094				
	$\overset{-}{x}$	0.052	0.127	0.090				
C22:1	Male	0.015	0.034	0.025	<0.05	1.000	0.898	0.003
	Female	0.015	0.035	0.025				
	$\overset{-}{x}$	0.015	0.034	0.025				
C24:1	Male	0.141	0.231	0.186	<0.05	0.911	0.630	0.007
	Female	0.138	0.237	0.187				
	$\overset{-}{x}$	0.140	0.234	0.187				
MUFA	Male	31.047 ^a^	28.489 ^b^	29.768	<0.05	0.251	<0.05	0.160
	Female	30.962 ^a^	28.767 ^b^	29.864				
	$\overset{-}{x}$	31.005	28.628	29.816				

s—species, g—sex, g x s—interaction sex x species, SEM—standard error of mean, MUFA—monounsaturated fatty acids, abc—means with different superscripts are significantly different at *p* ≤ 0.05.

**Table 5:** Polyunsaturated fatty acids profile (% total) of MS.

Fatty Acids	Sex	Species		$\overset{-}{x}$	*p*-Value			SEM
		Roe Deer	Red Deer		s	g	g x s	
C18:2 n-6	Male	11.690	12.463	12.076	<0.05	0.319	0.182	0.054
	Female	11.840	12.441	12.140				
	$\overset{-}{x}$	11.765	12.452	12.108				
C18:3 n-3	Male	3.986	3.325	3.656	<0.05	0.187	0.241	0.051
	Female	3.979	3.214	3.597				
	$\overset{-}{x}$	3.983	3.270	3.626				
C18:3 n-6	Male	0.053	0.141	0.097	<0.05	0.322	0.842	0.007
	Female	0.061	0.153	0.107				
	$\overset{-}{x}$	0.057	0.147	0.102				
C20:2	Male	0.071	0.046	0.059	<0.05	0.352	0.938	0.004
	Female	0.064	0.037	0.051				
	$\overset{-}{x}$	0.068	0.042	0.055				
C20:3 n-6	Male	0.357	0.349	0.353	0.301	0.920	0.162	0.012
	Female	0.322	0.379	0.351				
	$\overset{-}{x}$	0.340	0.364	0.352				
C20:3 n-3	Male	0.009 ^a^	0.050 ^b^	0.029	<0.05	<0.05	<0.05	0.004
	Female	0.053 ^b^	0.049 ^b^	0.051				
	$\overset{-}{x}$	0.031	0.049	0.040				
C20:4 n-6	Male	5.074	4.254	4.664	<0.05	0.944	0.944	0.055
	Female	5.071	4.254	4.663				
	$\overset{-}{x}$	5.073	4.254	4.663				
C20:5 n-3	Male	1.969	1.341	1.655	<0.05	0.546	0.687	0.047
	Female	1.980	1.394	1.687				
	$\overset{-}{x}$	1.975	1.367	1.671				
C22:2	Male	0.022	0.129	0.076	<0.05	<0.05	0.661	0.009
	Female	0.042	0.157	0.100				
	$\overset{-}{x}$	0.032	0.143	0.088				
C22:5 n-3	Male	1.951	1.298	1.624	<0.05	0.482	0.251	0.052
	Female	1.972	1.210	1.591				
	$\overset{-}{x}$	1.961	1.254	1.608				
C22:6 n-3	Male	0.372	0.046	0.209	<0.05	0.094	0.191	0.022
	Female	0.319	0.039	0.179				
	$\overset{-}{x}$	0.346	0.043	0.194				
PUFA	Male	25.555	23.442	24.498	<0.05	0.889	0.304	0.159
	Female	25.704	23.328	24.516				
	$\overset{-}{x}$	25.629	23.385	24.507				

s—species, g—sex, g x s—interaction sex x species, SEM—standard error of mean, PUFA—polyunsaturated fatty acids, ab—means with different superscripts are significantly different at *p* ≤ 0.05.

**Table 6:** Atherogenicity index (AI), thrombogenicity index (TI) and hypocholesterolaemic/hypercholesterolaemic ratio (HH) of *musculus semimembranosus.*

Indexes	Sex	Species		$\overset{-}{x}$	*p*-Value			SEM
		Roe Deer	Red Deer		s	g	g x s	
AI	male	0.439	0.717	0.578	<0.05	0.096	0.366	0.020
	Female	0.422	0.658	0.540				
	$\overset{-}{x}$	0.430	0.688	0.559				
TI	Male	0.731	1.016	0.873	<0.05	0.079	0.314	0.020
	Female	0.715	0.957	0.836				
	$\overset{-}{x}$	0.723	0.987	0.855				
HH	Male	2.582	1.655	2.118	<0.05	<0.05	0.167	0.061
	Female	2.630	1.832	2.231				
	$\overset{-}{x}$	2.606	1.743	2.175				

s—species, g—sex, g x s—interaction sex x species, SEM—standard error of mean.

## Data Availability

The data are available on request from the corresponding author.
